# Frequency of use and knowledge of the WHO-surgical checklist in Swiss hospitals: a cross-sectional online survey

**DOI:** 10.1186/1754-9493-7-36

**Published:** 2013-12-05

**Authors:** Anna C Mascherek, David LB Schwappach, Paula Bezzola

**Affiliations:** 1 Patient Safety Switzerland, Asylstrasse 77, 8032 Zurich, Switzerland; 2 Institute of Social and Preventive Medicine (ISPM), University of Bern, Finkenhubelweg 11, 3012, Bern, Switzerland

**Keywords:** Dissemination of surgical checklist, Self-rated knowledge, Objective knowledge, Satisfaction, Patient safety, Survey data

## Abstract

**Background:**

The WHO-surgical checklist is strongly recommended as a highly effective yet economically simple intervention to improve patient safety. Its use and potentially influential factors were investigated as little data exist on the current situation in Switzerland.

**Methods:**

A cross-sectional online survey with members (*N* = 1378) of three Swiss professional associations of invasive health care professionals was conducted in German, French, and Italian. The survey assessed use of, knowledge of and satisfaction with the WHO-surgical checklist. T-Tests and ANOVA were conducted to test for differences between professional groups. Bivariate correlations were computed to test for associations between measures of knowledge and satisfaction.

**Results:**

1090 (79.1%) reported the use of a surgical checklist. 346 (25.1%) use the WHO-checklist, 532 (38.6%) use the Swiss Patient Safety Foundation recommendations to avoid Wrong Site Surgery, and 212 (15.7%) reported the use of other checklists. Satisfaction with checklist use was generally high (doctors: 71.9% satisfied, nurses: 60.8% satisfied) and knowledge was moderate depending on the use of the WHO-checklist. No association between measures of subjective and objective knowledge was found.

**Conclusions:**

Implementation of a surgical checklist remains an important task for health care institutions in Switzerland. Although checklist use is present in Switzerland on a regular basis, a substantial group of health care personnel still do not use a checklist as a routine. Influential factors and the associations among themselves need to be addressed in future studies in more detail.

## Background

Patient safety has become an important issue worldwide since the Institute of Medicine published its article “To Err Is Human” in 1999 [1]. Since then, several interventions have been developed and established to improve patient safety [2-4]. Surgery is one major focus of health care improvement. In a recent, restrictive systematic review of studies on adverse events in surgery, Anderson at al. [5] found that adverse events occurred in 14.4% of patients. 5.2% of those events were judged as potentially preventable. Surgery has also been identified as a major predictor of patient reported hospital-acquired infection across 11 countries [6]. The World Health Organization’s (WHO) surgical checklist is an effective intervention to decrease morbidity and mortality in surgical procedures [7-9]. The checklist is now strongly recommended for adoption by international experts as a highly effective yet economically simple intervention [10].

However, implementation of a checklist alone does not necessarily lead to improvements in safety. Compliance with the checklist use is a prerequisite for the checklist to be effective [11]. Generally, compliance rates are often found to be below 100% [8,12-14]. Health care professionals’ (HCP) knowledge also plays a crucial role for correct and compliant checklist use [7,11,15,16]. Vats et al. [13] explicitly identified lack of knowledge as one important aspect for not correctly using the checklist. The better people are educated about how and why to use the surgical checklist, the more compliant they are. Both, subjective and objective levels of knowledge are important for changes in behaviour and thus successful implementation of interventions [17,18].

Shared knowledge and beliefs among HCPs are important for successful teamwork and cooperation and implementation of high safety standards, in particular in multiprofessional action-teams, like OR teams [12,19-24]. Recent research suggests that nurses often have more positive attitudes towards the checklist as compared to doctors and surgeons [22,23]. Thus, one essential prerequisite for successful checklist is the harmonization of checklist acceptance and knowledge among OR team members. Taken together, the effects of the use of surgical checklists on patient outcome have been widely acknowledged and relevant determinants for successful implementation have been identified. However, little data exist on the current situation in Switzerland. Available studies either address special settings [25] and samples (also see [26]) or are restricted to one hospital [14].

Hence, the aim of the present study was to evaluate checklist use and potentially influential factors in Switzerland. We investigated use, knowledge of, and satisfaction with the surgical checklist in Switzerland in a sample of different professional groups working together in the OR. Subjective and objective knowledge were both assessed as important factors influencing compliance. Levels of satisfaction were assessed, because we hypothesized that knowledge would not only be related to better compliance rates but also to higher levels of satisfaction.

## Methods

### Design

A cross-sectional online-survey study was conducted by the Patient Safety Foundation in Switzerland in December 2012. The EFS-Survey Software tool was used to program and administer the computer-based survey. The questionnaire was developed in German and then translated into French and Italian by professional translators. Translations were approved by HCP native speakers. The survey sample consisted of all members of three Swiss professional associations of invasive HCPs (doctors, nurses, nurses with special education in anaesthesia nursing or intensive care, and surgical technicians). Nurses were identified from the mailing list of all registered members of two professional associations. All doctors were members of the Foederatio Medicorum Chirurgicorum Helvetica, the holding organization of all sub-organisations of invasive specialties in Switzerland. Subjects were invited for participation by individual emails in which aim and procedure of the study were explained. Participants obtained a personal URL to the online-questionnaire and could then choose their preferred language (Italian, German, or French). Every URL could only be used once, hence, participation was only possible once per person. Two reminders were sent by mail with an interval of 1 week and 2 weeks, respectively to non-responders. Completion of the questionnaire was regarded as informed consent. Ethical approval is not necessary for this type of study in Switzerland.

### Survey

The survey was developed to assess use of, knowledge of and attitudes towards the WHO-surgical checklist. It was developed on the basis of extensive review of the literature. The survey consisted of three conceptual parts. The first part referred to the general use of surgical checklists in the OR (e.g., “which of the following checklists do you use?”) and relative frequency of use (rated on a 0-100% scale subdivided into 6 categories). Satisfaction with use was also assessed in part one and rated on a 5-point-Likert-scale ranging from “very unsatisfied” to “very satisfied”. In the second part, subjective and objective knowledge about the WHO-checklist was assessed. Subjects were presented 10 statements regarding the checklist (4 correct and 6 incorrect items) and were asked to indicate which of the items were correct or false. They rated their subjective knowledge about the checklist on a 5-point-Likert-scale from “very bad” to “very good”. In the third part, attitudes, norms and behavioural control towards checklist use [27,28] were assessed (not reported in this analysis). The survey was pre-tested with individuals from all professions and languages. Based on a professional translation, the survey was additionally validated by native speakers. The survey was not back translated.

### Data analyses

Survey data were analysed using descriptive statistics. For objective knowledge, a sum score was calculated based on the number of correctly answered questions out of 10. T-Tests and ANOVA were conducted to test for differences in frequency of use, satisfaction, and knowledge between professional groups. Bivariate correlations were computed to test for associations between measures of knowledge and satisfaction. All analyses were conducted using STATA v12.1 [29].

## Results

### Sample

Of the 5928 invited HCPs 1378 completed the survey (23.3% response rate). Sample characteristics are presented in Table 1.

**Table 1:** Sample characteristics by professional group in per cent*

		**Doctors**	**Nurses**
		***n*** **= 948**	***n*** **= 430**
Survey language	German	71.3	93.3
	French	25.7	6.1
	Italian	3.0	0.7
Gender	Female	22.5	67.2
	Male	77.5	32.8
Mean age in years (SD)		51.7 (7.8)	44.4 (9.1)
Education	Consultant	100	
	Theatre nurses		15.1
	Nurses with special education in		
	Anaesthesia nursing		77.2
	Intensive care		1.2
	Surgical technicians		6.5
Years of professional experience	0 – 2 years	1.0	3.7
	2 – 5 years	0.6	9.8
	5 – 10 years	4.3	16.7
	10 – 20 years	32.1	30.7
	More than 20 years	62.0	39.1
Percentage of time spent in the OR in an average week	Less than 10%	9.9	5.1
	10–25%	24.0	6.1
	26–50%	27.9	17.0
	51–75%	21.4	17.4
	78–90%	11.8	24.7
	91–100%	5.1	29.8
Primary workplace	Medical office	11.0	1.4
	Medical office and hospital	29.5	0.5
	Hospital	59.5	98.1
Type of hospital	University hospital	23.2	16.1
	Regional hospital with 500+ beds	17.6	18.3
	Regional hospital 125–499 beds	35.5	39.6
	Regional hospital up to 124 beds	13.7	16.6
	Paediatric clinic	0.5	1.4
	Other specialty	9.6	8.1

N = 1378.

*Age in years.

### Checklist use

**Figure 1:**
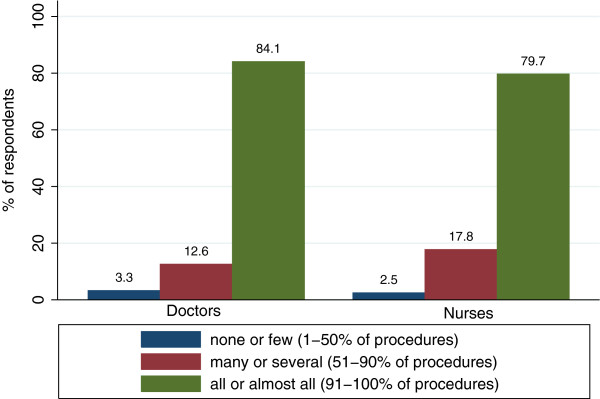
Frequency of checklist use at primary workplace for professional groups separately.

Among responders, 1090 (79.1%) reported the use of a surgical checklist in their OR. 346 (25.1%) participants use the WHO-checklist, 532 (38.6%) participants use the Swiss Patient Safety Foundation recommendations to avoid Wrong Site Surgery which is based on the Universal Protocol, and 212 (15.7%) reported the use of other checklists. 288 (20.9%) indicated to use no checklist at all. Addressing the frequency of use, Figure 1 shows that 84.1% of doctors and 79.7% of nurses who use a checklist use it virtually in all procedures. The difference in reported use between groups is not significant (*t*_(1088)_ = 1.18, n.s.; *M* = 5.7, *SD* = 0.8 for doctors, *M* = 5.7, *SD* = 0.7 for nurses).

### Satisfaction with checklist use

**Figure 2:**
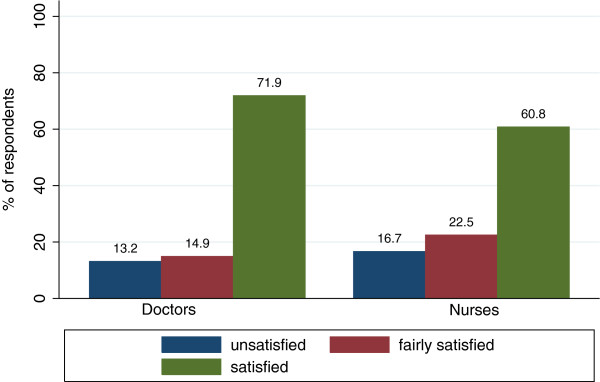
Satisfaction with checklist use at primary workplace for professional groups separately.

Satisfaction with checklist use was moderate (doctors: 71.9% satisfied, nurses: 60.8% satisfied). Figure 2 depicts satisfaction with checklist use at the primary workplace for both professional groups. Level of satisfaction of doctors (*M* = 2.6, *SD* = 0.7) is significantly higher compared to nurses (*M* = 2.4, *SD* = 0.8; *t*_(1088)_ = 3.1; *p* < .05).

### Knowledge regarding WHO-checklist

We report subjective and objective knowledge concerning the WHO-checklist, separately for individuals who work with the WHO-checklist at their primary workplace and those who do not (for wording of questions assessing objective knowledge see Table 2).

**Table 2:** Objective knowledge items

	**Question**	**Answer**
Question 1:	WHO-Checklist is a synonym for Team Time Out.	False
Question 2:	The WHO-checklist does not have to be signed by every member of the team.	Correct
Question 3:	The WHO-checklist asks for the exact documentation of the number of used swabs.	False
Question 4:	The WHO-checklist exclusively addresses surgeons.	False
Question 5:	The WHO-checklist recommends an antibiotic prophylaxis within 60 minutes before surgery.	Correct
Question 6:	The WHO-checklist shall support inexperienced members of the team.	False
Question 7:	The WHO-checklist is a tool used to attribute mistakes and misses to specific persons.	False
Question 8:	The WHO-checklist aims at preventing accidental omissions within routine procedures.	Correct
Question 9:	The WHO-checklist aims at improving team communication.	Correct
Question 10:	The WHO-checklist may be used to document complications.	False

On average, in the group of individuals who use the checklist, doctors answered 7.6 (*SD* = 1.4) and nurses 7.4 (*SD* = 1.2) out of 10 questions correctly. In the group of individuals who do not use the WHO-checklist, doctors, on average, responded to 7.4 (*SD* = 1.4) and nurses to 7.0 (*SD* = 1.5) out of 10 questions correctly. The results show that levels of knowledge were significantly different between doctors and nurses as well as between individuals working with the checklist and those who do not (professional group: F(1;625) = 4.4; p < .05, WHO-checklist use: F(1;625) = 8.2; p < .05). Note, however, that both variables explained only about 2% of variance. The practical relevance of the differences is small.

For subjective knowledge, different results emerged. In the group of individuals not working with the checklist, 98.4% of the doctoral staff and 97.8% of the nursing staff evaluated their knowledge as being bad. In the group working with the checklist, almost the opposite picture emerged: 80.2% of the doctoral staff and 75.8% of the nursing staff evaluated their knowledge as being good. Differences were statistically significant only between users of the WHO-checklist and non-users (WHO-checklist use: F(1;625) = 106.7; p < .05). For subjective knowledge, WHO-checklist use explained about 18% of variance. Hence, working with the WHO-checklist was pivotal for self-rated knowledge in both groups. For objectively assessed knowledge, both, WHO-checklist use and professional group affiliation significantly influenced the outcome.

In a last step of the analyses, associations between subjective and objective knowledge and satisfaction with checklist-use were examined for checklist use separately. Results for the bivariate correlations are depicted in Table 3.

**Table 3:** Correlations between self-rated knowledge, objective knowledge, and satisfaction for professional groups separately in WHO-checklist users

	**Objective knowledge**	**Self-rated knowledge**
Doctors		
Self-rated knowledge	0.09	
Satisfaction	-0.02	0.12
Nurses:		
Self-rated knowledge	-0.1	
Satisfaction	0.09	0.16

Note: None of the correlations were statistically significant.

As can be seen from Table 3, the correlations between satisfaction, objective knowledge and subjective knowledge are very small. None of the tested correlations were statistically significant.

## Discussion

In the present study we investigated the use and knowledge of the WHO surgical checklist in Switzerland. To the best of our knowledge, the study is the first providing a comprehensive overview including all parts of Switzerland as well as different professional groups working together in the OR.

The majority of participants reported the systematic use of a surgical checklist at their primary workplace with no significant differences between the professions. In their study, Fourcade et al. report compliance rates about 92%, however with completion rates being as low as 61% [8,12]. In their systematic literature review, Borchard et al. report compliance rates ranging from 12% to 100% [11]. Hence, although the results from the present study are comparable to results from the literature, checklist use is still no routine and improvement is needed. Literature shows that one crucial aspect of improving patient safety with the use of a surgical checklist is 100% compliance, including, urgent surgeries [11,30]. Hence, from the present study the conclusion seems warranted that even though surgical checklists seem to be widely used by some professional groups in Switzerland, still effort and time need to be invested to establish a reliable and exhaustive use for all procedures.

Overall, both professional groups reported moderate satisfaction. This indicates that in ORs where the checklist has been implemented, acceptance is present. But satisfaction with checklist use is likely to be overestimated in the present study. Considering participants’ professional position and years of job experience we infer that individuals in the sample hold influential positions and probably have the opportunity to shape organizational structures and standards. Still up to 16% of nurses and 13% of doctors reported being unsatisfied. One may conclude that individuals in less influential positions might report even lower levels of satisfaction. It is known from psychological research, that perceived control is associated with higher levels of satisfaction (see for example [31]). Hence, one might infer, that individuals in influential positions experience higher levels of control, and, hence might report higher levels of satisfaction. However, this remains speculative and future studies need to investigate predictors of satisfaction with checklist in more detail. Significant differences in levels of satisfaction emerged between professional groups. Doctors reported higher levels of satisfaction than nurses. Group differences in the OR are known from other OR-relevant topics, e.g. perception of teamwork [19,22,24]. The origin of these differences remains unclear.

In the analyses of knowledge regarding the WHO-checklist, we found significant differences between WHO-checklist users and non-users and a difference between professional groups. Individuals working with the WHO-checklist achieved significantly higher levels of knowledge than individuals not working with the WHO-checklist. Although doctors achieved higher levels of knowledge than nurses, the effect was too small to be of practical relevance. Note that no inferences about causality can be made. Correlations reflect associations between variables. However, a correlation between variables does not imply that one causes the other. The reason for or the direction of the association between variables is not explained by correlations. Hence, whether better knowledge leads to checklist use or checklist use leads to better knowledge remains unclear.

In a last step, the association between subjective knowledge, objective knowledge, and satisfaction was assessed for both professional groups. No significant association could be found between any of the constructs in both groups. It is known from social cognitive research that not only objectively assessable knowledge but also subjective levels of knowledge are an important factor for the evaluation of situations, attitudes and behaviour [32]. Subjective representations are often of behavioural relevance (e.g., [33] on aging). As a consequence, subjective evaluation of one’s own expertise can be decisive for developing the motivation to take part in trainings or further education concerning the correct use of checklists (e.g.,[34]). With no association between the constructs, as found in the present study, the possibility of a gap between needed and claimed education and training or an overly excessive use of education increases.

This result has important implications for practice: with no association between self-evaluation and objective assessment, employees cannot reliably self-elect themselves for training when needed, because of the lack of adequate assessment of their own level of expertise. In case of overestimation this imposes a possible threat on patients, in case of underestimation, this leads to a waste of resources. The importance of organizational commitment in the process of checklist implementation and the promotion of compliance becomes evident. Without reliable self-management due to a lack of valid self-evaluation, an organization is in charge of ensuring that employees obtain training and education on a regular, institutionalized basis to improve patient safety. Note, however, because subjective knowledge was assessed with one item only, the effect of measurement error also needs to be considered.

The present study has some limitations. First we focused on the WHO-checklist in survey items. The assessment of use and knowledge of other checklists may be underestimated. Because surgical checklists contain similar items and have similar objectives, individuals working with other checklists might have profound knowledge about checklists that has not been assessed in the present study, yet is an important aspect of patient safety in surgery as well. Second, although we assessed compliance in terms of the frequency of use, we have no information about the quality of use. Using the checklist does not exhibit any information about the quality of use as the study by Cullati et al. indicates [14]. Third, our response rate was only moderate. Quantitatively, this response rate is in accordance with similarly designed studies in health care [35]. Although different professional groups and geographical regions are well represented in our sample, we have no information about reasons for non-participation and checklist use among non-responders. However, it is likely that HCP working with an interest in patient safety or the surgical checklist were more likely to respond to our survey. Thus, our results probably overestimate checklist dissemination in Switzerland.

## Conclusion

Taken together, what do the results of the present study say about frequency of use, knowledge of, and satisfaction with the WHO-surgical checklist in Switzerland?

According to the results of the present study, checklist use is present in Switzerland on a regular basis. However, a substantial group of HCP’s still does not use checklists as a routine. Satisfaction with checklist use is moderate. Improvement of satisfaction could be used as motivation to fully and comprehensively implement the checklist. Additionally, against the background that knowledge is crucial for successful implementation, levels of knowledge in the present study reveal need for improvement. Even for individuals working with checklists, levels of knowledge are far from perfect. Differences between groups indicate that knowledge acquisition is not systematically supported but is left to individuals. Much more attention needs to be paid to staff’s training and education concerning checklist use.

We conclude that the results provide valuable insight into checklist use in Switzerland.

## Competing interests

The authors declare that they have no competing interests.

## Authors’ contribution

AM and DS wrote the manuscript. AM conducted statistical analyses. DS and PB designed the study and supervised the research. All authors read and approved the final manuscript.
